# Stimulus Presentation at Specific Neuronal Oscillatory Phases Experimentally Controlled with tACS: Implementation and Applications

**DOI:** 10.3389/fncel.2016.00240

**Published:** 2016-10-18

**Authors:** Sanne ten Oever, Tom A. de Graaf, Charlie Bonnemayer, Jacco Ronner, Alexander T. Sack, Lars Riecke

**Affiliations:** ^1^Department of Cognitive Neuroscience, Faculty of Psychology and Neuroscience, Maastricht UniversityMaastricht, Netherlands; ^2^Department of Engineering and Instrumentation, Faculty of Psychology and Neuroscience, Maastricht UniversityMaastricht, Netherlands

**Keywords:** tACS, oscillations, phase, brain stimulation, stimulus presentation

## Abstract

In recent years, it has become increasingly clear that both the power and phase of oscillatory brain activity can influence the processing and perception of sensory stimuli. Transcranial alternating current stimulation (tACS) can phase-align and amplify endogenous brain oscillations and has often been used to control and thereby study oscillatory power. Causal investigation of oscillatory phase is more difficult, as it requires precise real-time temporal control over both oscillatory phase and sensory stimulation. Here, we present hardware and software solutions allowing temporally precise presentation of sensory stimuli during tACS at desired tACS phases, enabling causal investigations of oscillatory phase. We developed freely available and easy to use software, which can be coupled with standard commercially available hardware to allow flexible and multi-modal stimulus presentation (visual, auditory, magnetic stimuli, etc.) at pre-determined tACS-phases, opening up a range of new research opportunities. We validate that stimulus presentation at tACS phase in our setup is accurate to the sub-millisecond level with high inter-trial consistency. Conventional methods investigating the role of oscillatory phase such as magneto-/electroencephalography can only provide correlational evidence. Using brain stimulation with the described methodology enables investigations of the *causal* role of oscillatory phase. This setup turns oscillatory phase into an independent variable, allowing innovative, and systematic studies of its functional impact on perception and cognition.

## Introduction

Brain activity around the time of stimulus presentation can determine processing efficacy. Human research with magneto-/electroencephalography (M/EEG) demonstrated this by *post-hoc* correlation of perceptual/cognitive task performance to measures of oscillatory power (Pfurtscheller, 1981; Tallon-Baudry and Bertrand, 1999; Jensen and Mazaheri, 2010) or phase (Mathewson et al., 2010; Fiebelkorn et al., 2013; Ten Oever et al., 2015). Oscillatory phase is currently believed to act as a “sensory gatekeeper” (Buzsáki, 2004; Schroeder and Lakatos, 2009; Giraud and Poeppel, 2012) in local and long-range neuronal communication (Singer and Gray, 1995; Engel and Singer, 2001; Fries, 2005), but its functional role is still poorly understood. Overall, M/EEG phase studies are highly informative, but conceptually limited by the correlational nature of their approach and methodologically constrained by the *post-hoc* nature of the phase analysis.

“Entrainment” approaches (Thut et al., 2011; Herrmann et al., 2015) go one step further, transforming oscillatory brain activity into an independent variable (Sack, 2006; Herrmann et al., 2015), for instance using transcranial alternating current stimulation (tACS; Antal et al., 2008; Kanai et al., 2008; Feurra et al., 2011). In tACS, a low-intensity electrical current passes between two or more electrodes placed on the scalp, periodically switching direction at an externally controlled frequency. This entrainment approach allows *causal* investigations of neuronal oscillations and has been successfully used to study the functional role of oscillatory power (Antal et al., 2008; Kanai et al., 2008; Zaehle et al., 2010; Feurra et al., 2011; Strüber et al., 2014; Dowsett and Herrmann, 2016). However, the use of tACS for studying the causal role of oscillatory phase is currently limited (but see Neuling et al., 2012; Riecke et al., 2015a,b; Raco et al., 2016), perhaps because the presentation of sensory stimuli at experimentally controlled oscillatory phases is technically challenging. Yet, oscillatory phase-based stimulus presentation is a key prerequisite for going beyond correlational magneto-/electroencephalography (M/EEG) phase studies.

Here, we present and validate custom software and hardware solutions that provide experimenters full simultaneous temporal control over multiple stimuli in various modalities (electrical/sensory/magnetic). The proposed approach enables the presentation of stimuli at any desired phase of the entraining tACS signal. To the extent that tACS can entrain neuronal oscillatory phase, this implies that stimuli can be introduced at desired neuronal oscillatory phases. Moreover, since our setup allows simultaneous control in multiple modalities and stimulation devices, it opens up a wide range of research applications.

## Materials and methods

### Setup overview

Our experimental setup contains several commercially available hardware components, and revolves around a custom-developed standalone software solution, “DataStreamer,” that we make freely available here:

<<https://osf.io/h6b8v/>>

The downloadable materials include, along with our DataStreamer software, example, and template files for creating stimulus protocols and a comprehensive user guide. The authors can be contacted for support.

**Figure 1:**
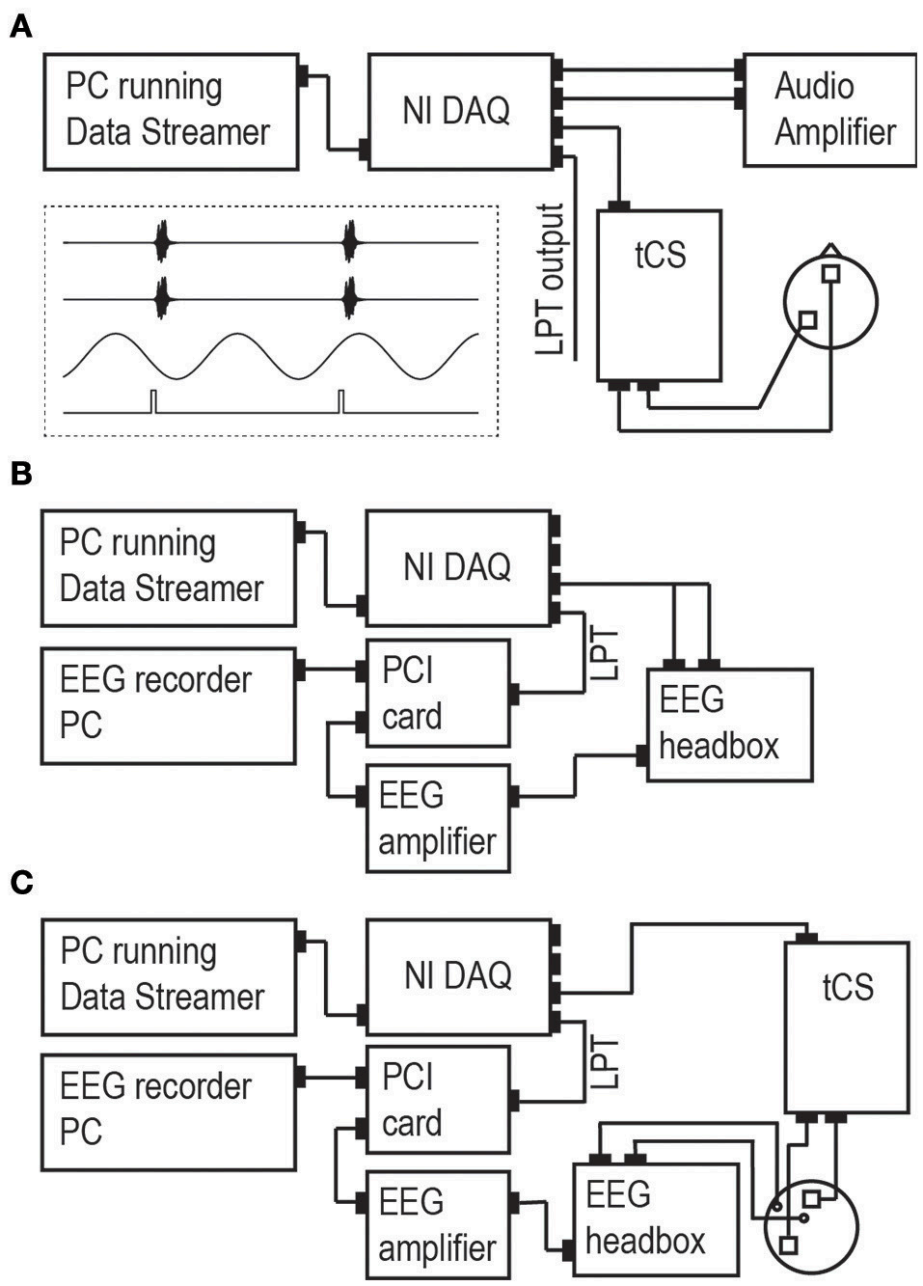
**Set-up. (A)** The main set-up consists of a PC running our custom software (“Data Streamer”). This computer is connected to a multi-channel NI DAQ that converts the digital time series into analog/digital output signals and keeps all output streams synchronized. NI DAQ channel output is sent to a tCS device, to an auditory amplifier (in the case of auditory experiments), and/or as an LPT trigger to present visual, somatosensory, or magnetic stimuli through preferred stimulation devices. Example stimulus protocols for the Data Streamer in an auditory experiment are given in the inset (two auditory streams in channels 1/2, tACS stream in channel 3, LPT triggers connected to stimulus presentation PC in channel 4). The auditory amplifier could be any other stimulation device accepting analog signals as input. Panels **(B,C)** display the setup for experiment 1 and 2, respectively. Experiment 1 assessed the validity of the setup up to and including the stage of the NI DAQ. Experiment 2 added a tCS device and a head model (a melon), enabling to assess the validity of the complete setup.

Figure 1A illustrates the design of our setup. Its general working principle is as follows: The experimenter designs per participant and per experimental run a *stimulus protocol* in the form of a digital file. The stimulus protocol file contains all the tACS information and stimulus information, including their relative timing, in the form of multiple time series (representing the tACS signal, stimulus triggers, and other stimuli; see inset in Figure 1A). The protocol is loaded into DataStreamer, which allocates the different time series to separate channels of a National Instruments digital-to-analog converter (*NI DAQ*). The NI DAQ feeds these signals to relevant connected devices serving stimulus presentation (tCS machines, stimulus computers, or other stimulation devices). In the following sections, we provide more details on the setup and procedures, as well as the Section Materials and Methods for our validation experiments reported under Section Results.

### Software and hardware

*Stimulus protocols* are generated digitally prior to the experiment. Each time series in the protocol represents a specific stimulation channel either as a *continuous waveform* (for time-varying signals such as tACS or auditory/somatosensory stimuli) or as *discrete on/off pulses* (e.g., for LPT *triggers*), all sampled at a common, experimenter-defined sampling rate. The stimulus protocols are coded collectively in a single 2-D matrix (with stimulation channels in rows and sampling points in columns); for a visual representation of an example matrix, see inset in Figure 1A.

Our DataStreamer software, which we developed using LabVIEW 2014 (National Instruments, Austin, TX), controls external stimulation devices as specified by the pre-generated stimulus protocols. While it currently accepts stimulus protocols (2-D matrices) stored in.MAT data format [Matlab (MathWorks, Natick, MA)], an updated version accepting text files (.TXT) will be made freely available upon request. DataStreamer feeds the time series from the imported stimulus protocol file—continuously and in parallel—as data chunks of desired size via USB into a multi-channel NI DAQ. The NI DAQ converts the multiple data streams in parallel into multiple signals, which are subsequently fed into multiple external stimulation devices (e.g., sound card, video card, audio amplifier, or one or more tACS/TMS systems).

The NI DAQ *output waveforms* can be utilized to directly control the shape and timing of stimulation (e.g., tACS signals or auditory signals) provided that the external stimulation device allows external control of stimulation voltage, as for example the NeuroConn tCS stimulator with “Remote” option (Ilmenau, Germany) used in our lab.

The discrete NI DAQ *output pulses* can be used to trigger (i.e., control the timing of) externally generated stimuli. These stimuli can take any form or modality, depending on the connected external stimulation device [e.g., a standard PC serving sensory stimulus presentation, or other stimulation devices equipped with pre-installed signal waveforms, such as transcranial magnetic stimulation (TMS) machines]. Although the output pulses act as instantaneous triggers, they can still take multiple values, thereby enabling the implementation of multiple conditions within the stimulus protocol. For instance, in a visual experiment with two possible stimulus locations, the stimulus PC can identify the current condition based on the value of the incoming trigger, and present a stimulus in the appropriate location on screen. In short, the PC would immediately present a stimulus, but with parameters that depend on the trigger value.

An important caveat associated with external stimulation hardware is that the actual timing of stimulus presentation will depend on response parameters of the external devices that are used. As soon as peripheral hardware receives a trigger, it will provide the requested response (i.e., a specific stimulus) at its earliest opportunity. But that earliest opportunity can differ widely for different devices. For instance, a LED device directly receiving a trigger might add virtually no temporal offset in its light emission response. But stimuli to be presented on a standard digital computer monitor would appear on screen with a certain (knowable) delay, depending on monitor refresh rate, monitor response time, screen location of requested stimulus, and the timing of the incoming trigger in relation to the monitor's refresh cycle. These are not limitations of the setup proposed here, but limitations inherent to external stimulation hardware that should be kept in mind when designing experiments and selecting stimulation devices.

A key advantage of our setup is that it provides precise and simultaneous control over multiple stimulation channels, thereby enabling multisensory stimulation of any kind (e.g., tACS, TMS, auditory/visual/somatosensory stimuli), while at the same time providing full freedom and full control over conditions and parameters. The total number of external stimulation devices that can be controlled in parallel depends on the number of available NI DAQ channels (four on the NI USB-6343 DAQ used in our lab).

### Validation measurements

We performed extensive measurements to assess the precision and validity of our setup. The most relevant channels for studying causal phase effects are those carrying output waveforms (e.g., the tACS signal; see inset in Figure 1A, third row) and output pulses (e.g., triggers for temporally precise presentation of pre-defined sensory stimuli; see inset in Figure 1A, fourth row). We conducted two experiments in which we recorded these specific channels at two different stages of the signal pathway using a calibrated EEG system.

Connectivity schemes for both experiments are depicted in Figures 1B,C. In *experiment 1*, we directly measured the output of the NI DAQ, by feeding the analog waveform (“tACS signal”) via a custom-made cable into the EEG headbox and the discrete pulses (triggers) via LPT connection into a peripheral component interface (PCI) adaptor card which feeds both the EEG and the LPT trigger to a recording PC. The pulses appear thereby as markers in the EEG recording. EEG was recorded using a standard EEG system (BrainAmp, BrainProducts GmbH, Munich, Germany) and software (BrainVision Recorder). EEG recordings were lowpass-filtered in the analog domain (cutoff frequency: 250 Hz) and then digitized (sampling rate: 5000 Hz). All other online filters (except for the 250 Hz high cut-off) were switched off and no offline filters were applied in any of the analyses. If our setup is valid, these measurements should reveal only negligible inaccuracies in stimulus timing induced by the measurement apparatus (i.e., a delay of the analog waveform relative to the triggers within the sub-millisecond range), because triggers were fed directly into the recording PC whereas waveforms underwent some additional processing (amplification and analog-to-digital conversion).

In *experiment 2*, we measured the output of the entire signal pathway by connecting the NI DAQ to a tCS device, applying the tACS to a melon [two tACS electrodes of 5^*^5 cm (NeuroConn, Ilmenau, Germany) applied with conductive gel (Ten20®, DO Weaver, Aurora, CO, USA)], and measuring with two EEG electrodes the electric potentials induced in this head model. The EEG recording electrode was positioned close to a tACS electrode and the EEG reference electrode positioned further away. We predicted that the addition of the tCS apparatus (tCS device, tCS electrodes, and conductive paste) and conductive head model (including EEG electrodes and conductive paste) would cause additional inaccuracies relative to experiment 1 that are still of an acceptable order of magnitude.

We tested a range of phases and frequencies in both experiments. Specifically, for tACS frequencies of 5, 10, 20, 40, and 80 Hz, we presented triggers at five phases each, equally spaced and covering one full period of the respective waveform. Per phase and per frequency, we presented 50 trials (lasting 2.5 min). We used intensities ranging from −25 to 25 mV, which was converted by the tCS device to an output of −0.05 to 0.05 mA.

### Analysis

EEG signals and trigger markers were analyzed using the FieldTrip toolbox (Oostenveld et al., 2011), the circular statistics toolbox (Berens, 2009), and custom Matlab scripts (MathWorks). Per trial (i.e., LPT trigger, recorded as EEG marker), the phase of the oscillating waveform was determined using the Hilbert transform, extracting the instantaneous phase at the onset of the trigger (function *ft_preprocessing* in Fieldtrip Oostenveld et al., 2011). Results are presented in circular phase plots. Across trials, event-related potentials over 2-s epochs, time-locked to each of the five phase-condition triggers (see insert in Figure 1A, fourth line of stimulus protocol, communicated through LPT signals), were created to further visualize the temporal correspondence of LPT triggers to the oscillating waveform at different phase conditions.

The primary measures of interest were *absolute phase shift* and *phase consistency*. We define *absolute phase shift* as the difference between the observed phase (the measured phase of triggers respective to the measured oscillatory waveform) vs. the desired phase (the requested phase of triggers in the stimulus protocol respective to the oscillatory time series in the stimulus protocol). We used this measure to assess the accuracy of our setup. We further assessed the reliability of our setup based on *phase consistency*, which we define as the range of observed phases across trials around the mean observed phase. We use two specific measures of phase consistency: the *maximum offset* (i.e., the largest observed difference between observed phase and mean observed phase) and the *95th percentile offset* (the 95th percentile of the aforementioned differences). The latter thus shows how consistent phase offset is for the vast majority of trials, leaving out the 5% most extreme trials. In other words, 95% of all trials yielded phases closer to the observed mean phase than the 95th percentile offset. We calculated these offsets in terms of phase (degrees) and in terms of time (milliseconds).

## Results

### Experiment 1

**Figure 2:**
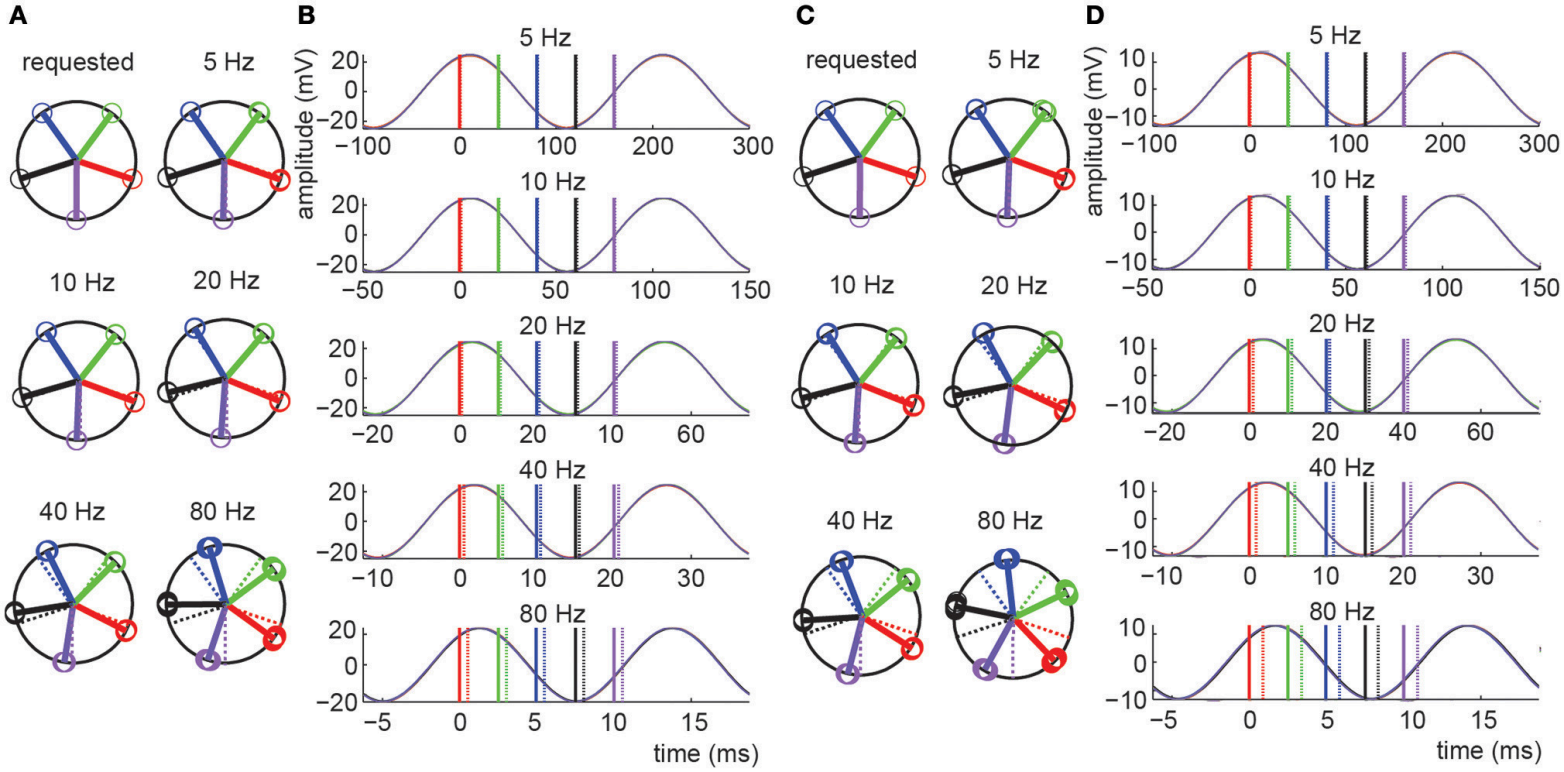
**Results for experiments 1 and 2**. **(A,C)** Circular phase plots presenting the phase of the tACS sinusoid at which LPT triggers were measured for experiment 1 **(A)** and experiment 2 **(C)**. The “requested” plot is based on the stimulus protocol file, thus representing the target trigger-tACS phases. Actually measured trigger-tACS phases are presented in the additional plots labeled by the frequency condition they reflect. In these plots, dashed colored lines reproduce the target phases, for easy comparison. Different phase conditions are presented in different colors (blue, green, red, purple, black, for subsequent phases), open circles present individual measurements, solid lines present the circular mean phase for all trials in each phase condition. A consistent absolute phase shift in the measurements, expressed in milliseconds, is reflected by a phase shift in degrees that increases with frequency. Phase consistency (stability of the measured phase across trials) is very high as evidenced by the fact that individual datapoints (open circles) essentially fully overlap (see Tables for numerical presentations). **(B,D)** Event related potentials (ERPs) of the different phases and frequencies tested in experiments 1 **(B)** and experiment 2 **(D)**. The ERPs are aligned to the average phase of the first phase condition. Solid and dashed lines represent the average measured phase (relative to the first bin) and the requested phase respectively. Note that in the time domain, the observed difference between requested and measured phase was on average only 0.52 ms for experiment 1 and constant across frequencies (note the different time scales in the ERP plots). This delay was 0.94 ms for experiment 2.

In experiment 1 we directly assessed the temporal relation between waveform and pulse outputs of the NI DAQ. Results are shown in Figure 2 and Tables 1–3, and confirm that *absolute phase shift* and *phase consistency* were highly accurate. The absolute phase shift can easily be evaluated from Figure 2, which visualizes the equal spacing of the five requested phase conditions as well as the measured phases for those conditions, in both the phase domain (circular phase plots) and time domain (event-related potential plots). The mean absolute phase shift (the difference between mean observed phase and mean requested phase) shown in Tables 1, 3 is in the sub-millisecond range, with measured triggers slightly preceding the measured waveforms, as predicted. Note that, although absolute phase shift (expressed in units of degrees) rises with frequency, time-domain analysis reveals that its duration (expressed in units of ms) is consistent across both trials and frequencies tested (e.g., **Table 3**) and only of negligible magnitude (~0.5 ms).

**Table 1:** **Absolute phase shift**.

	**Phase bin 1**		**Phase bin 2**		**Phase bin 3**		**Phase bin 4**		**Phase bin 5**	
	**Deg**	**ms**	**Deg**	**ms**	**Deg**	**ms**	**Deg**	**ms**	**Deg**	**ms**
**EXPERIMENT 1**										
5 Hz	0.96	0.54	0.82	0.45	1.01	0.56	0.97	0.54	1.02	0.57
10 Hz	1.93	0.54	1.90	0.53	1.96	0.54	1.81	0.50	1.97	0.55
20 Hz	3.80	0.53	3.79	0.53	3.81	0.53	3.67	0.51	3.73	0.52
40 Hz	7.59	0.53	7.61	0.53	7.36	0.51	7.43	0.52	7.38	0.51
80 Hz	15.25	0.53	14.57	0.51	14.87	0.52	14.50	0.50	15.54	0.54
**EXPERIMENT 2**										
5 Hz	1.94	1.08	1.64	0.91	1.88	1.05	1.78	0.99	1.84	1.02
10 Hz	3.43	0.95	3.36	0.93	3.56	0.99	3.34	0.93	3.46	0.96
20 Hz	6.74	0.94	6.69	0.93	6.69	0.93	6.62	0.92	6.57	0.91
40 Hz	13.17	0.91	13.18	0.92	13.04	0.91	13.16	0.91	13.01	0.90
80 Hz	25.46	0.88	25.58	0.89	25.38	0.88	26.23	0.91	25.79	0.90

*The table shows per requested phase (phase bin), per experiment (1 and 2), the absolute phase shift (difference between requested and mean observed phase) in degrees and transformed to the time domain in milliseconds*.

Phase consistency is numerically reported in Tables 2, 3. In Table 2, the ranges of observed phase offsets are displayed, both the maximum offset and the 95th percentile offset (see Section Materials and Methods for details). Table 3 presents these results collapsed over phase bin conditions. From Table 3 it is evident that 95% of the observations in all conditions displayed temporal offsets within 0.13 ms (i.e., 95% of trials yielded a trigger phase within 0.13 ms of the mean phase), so phase consistency was well within the sub-millisecond range for the vast majority of trials. When including all observations the maximum inaccuracy was 1.07 ms.

**Table 2:** **Phase consistency**.

	**Phase bin 1**		**Phase bin 2**		**Phase bin 3**		**Phase bin 4**		**Phase bin 5**	
	**Maximum offset**		**Maximum offset**		**Maximum offset**		**Maximum offset**		**Maximum offset**	
	**Deg**	**ms**	**Deg**	**ms**	**Deg**	**ms**	**Deg**	**ms**	**Deg**	**ms**
**EXPERIMENT 1**										
5 Hz	1.93	1.07	1.71	0.95	0.29	0.16	0.19	0.10	0.30	0.17
10 Hz	0.46	0.13	0.35	0.10	0.41	0.11	0.37	0.10	0.38	0.11
20 Hz	0.79	0.11	0.80	0.11	0.73	0.10	0.82	0.11	0.73	0.10
40 Hz	1.44	0.10	1.55	0.11	1.58	0.11	1.46	0.10	1.45	0.10
80 Hz	3.27	0.11	3.08	0.11	2.82	0.10	2.90	0.10	3.44	0.12
**EXPERIMENT 2**										
5 Hz	1.95	1.08	3.54	1.97	0.59	0.33	0.57	0.32	0.64	0.36
10 Hz	1.71	0.48	1.37	0.38	1.41	0.39	0.87	0.24	1.05	0.29
20 Hz	1.67	0.23	1.24	0.17	1.19	0.17	1.49	0.21	1.53	0.21
40 Hz	1.76	0.12	3.20	0.22	1.86	0.13	1.77	0.12	2.37	0.16
80 Hz	3.88	0.13	3.35	0.12	3.25	0.11	4.54	0.16	3.33	0.12
	**Phase bin 1**		**Phase bin 2**		**Phase bin 3**		**Phase bin 4**		**Phase bin 5**	
	**95th percentile offset**		**95th percentile offset**		**95th percentile offset**		**95th percentile offset**		**95th percentile offset**	
	**Deg**	**ms**	**Deg**	**ms**	**Deg**	**ms**	**Deg**	**ms**	**Deg**	**ms**
**EXPERIMENT 1**										
5 Hz	0.24	0.14	0.26	0.14	0.23	0.13	0.15	0.09	0.23	0.13
10 Hz	0.42	0.12	0.30	0.08	0.37	0.10	0.29	0.08	0.32	0.09
20 Hz	0.71	0.10	0.71	0.10	0.71	0.10	0.78	0.11	0.62	0.09
40 Hz	1.43	0.10	1.28	0.09	1.36	0.09	1.34	0.09	1.37	0.09
80 Hz	2.70	0.09	2.67	0.09	2.66	0.09	2.64	0.09	3.23	0.11
**EXPERIMENT 2**										
5 Hz	0.66	0.36	0.74	0.41	0.52	0.29	0.49	0.27	0.51	0.28
10 Hz	0.77	0.21	0.84	0.23	0.89	0.25	0.76	0.21	0.89	0.25
20 Hz	1.22	0.17	0.97	0.13	0.88	0.12	0.86	0.12	1.14	0.16
40 Hz	1.50	0.10	1.88	0.13	1.54	0.11	1.54	0.11	1.62	0.11
80 Hz	3.08	0.11	2.75	0.10	2.82	0.10	3.29	0.11	3.02	0.10

*Separately for frequency and phase conditions, per experiment, columns present the consistency of trigger phases as the maximum offset (top part of graph) in degrees and milliseconds. Since these ranges are determined by rare outliers, a useful additional measure is phase consistency across the majority of trials. The 95th percentile of the offsets across trials presented here in milliseconds (bottom part)*.

**Table 3:** **Overview: results collapsed over phase bins**.

	**Absolute phase shift**		**Phase consistency: maximum offset**		**Phase consistency: 95th percentile offset**	
	**Deg**	**ms**	**Deg**	**ms**	**Deg**	**ms**
**EXPERIMENT 1**						
5 Hz	0.96	0.53	1.93	1.07	0.23	0.13
10 Hz	1.91	0.53	0.46	0.13	0.36	0.10
20 Hz	3.76	0.52	0.82	0.11	0.71	0.10
40 Hz	7.47	0.52	1.58	0.11	1.37	0.09
80 Hz	14.95	0.52	3.44	0.12	2.68	0.09
**EXPERIMENT 2**						
5 Hz	1.82	1.01	3.54	1.97	0.59	0.33
10 Hz	3.43	0.95	1.71	0.48	0.86	0.24
20 Hz	6.66	0.93	1.67	0.23	1.13	0.16
40 Hz	13.11	0.91	3.20	0.22	1.62	0.11
80 Hz	25.69	0.89	4.54	0.16	3.02	0.10

*Results from Tables 1, 2 are shown here collapsed over phase bin conditions. Columns display in degrees and milliseconds the absolute phase shift and phase consistency*.

### Experiment 2

In experiment 2 we applied tACS to a head model and measured EEG. The addition of the tCS device, electrode montage, and conductive head model, only slightly increased the measured absolute phase shift. Translated to the time-domain, the absolute phase shift was on average 0.41 ms longer than in experiment 1 (Table 3). Furthermore, while phase consistency slightly decreased, it remained very high, with 95% of phase offsets falling within 0.33 ms of the mean phase (maximum offset was 1.97 ms). It seems that the tCS device introduced a frequency-dependent shift in both absolute phase shift and phase consistency, such that higher frequencies resulted in a lower mean phase shifts and higher phase consistency, in milliseconds (see Table 3). However, these cross-frequency differences were again negligible (all falling well within the sub-millisecond range) and therefore unlikely to influence commonly applied neuroscientific set-ups.

## Discussion

Our validation measurements demonstrated that our proposed setup enables presentation of stimuli accurately at specific phases of the tACS oscillatory cycle, with high inter-trial consistency, in near-perfect relative phase relations, and with only a small delay in terms of absolute phase. To summarize, the setup crucially includes:

- A National Instruments digital-to-analog converter (commercially available)
- tCS apparatus with an external voltage control option (commercially available), our setup was tested with NeuroConn devices with “Remote” option
- DataStreamer software (now freely available with user guide and templates)
- Stimulus protocols, created by the experimenter, including tACS and other stimuli and/or stimulus triggers, in.MAT file format (or.TXT, see Section Materials and Methods).

Other labs have successfully achieved tACS setups (Helfrich et al., 2014; Dowsett and Herrmann, 2016) with phase-dependent stimulus presentation (Neuling et al., 2012; Raco et al., 2016). Here, we provide full details on the accuracy and procedure of our specific setup, which allows full, simultaneous, independent, and precise control of sensory stimulation in relation to tACS phase. *Full*, because the stimulation protocol can fully be predetermined for an entire experimental run. *Simultaneous*, because all stimulation devices (or channels of individual devices) can be controlled in parallel, allowing multisensory stimulation. *Independent*, because each of these devices or channels is freely controllable without mutual constraints. *Precise*, because all stimulation devices (or channels) including tCS are controlled by a common clock with high sampling rate (up to 500 kHz for our specific DAC), resulting in stable stimulus timings as shown by our validation measurement results. Potential delays introduced by external stimulation devices should be considered on a case basis.

All hardware in our setup is commercially available and our custom software is freely available (link provided in the Materials and Methods Section). Critical features of the setup are that the NI DAQ needs to provide a number of channels equaling at least the overall number of independent stimulation channels to be used in planned experiments, and the ability of available stimulators (e.g., tCS system) to be controlled remotely by a signal generator. The setup is applicable not only to tACS plus additional stimulation, but also to TMS, multisensory stimulation, intracranial AC stimulation, or other implementations requiring precise timing control over multiple stimulation devices. It is therefore a very flexible setup that can provide solutions for a wide range of applications even beyond tACS.

When compared to existing solutions, DataStreamer has a few advantages. The software is user-friendly and freely available. Support is available in terms of supplied documentation (comprehensive user guide, file templates that can be easily adapted to different experiments) and contact with the authors. In terms of performance, our validation measurements suggested that it performed minimally as well as the Matlab DAQ toolbox, the only other available software that we are currently aware of (See Supplementary Materials for Figures and data of our measurements with this software).

The tACS phase-based stimulus presentation approach makes it possible to apply powerful experimental designs that cannot be easily implemented in conventional M/EEG studies of oscillatory phase. Bringing oscillatory phase under control (i.e., transforming it into an independent variable) provides the following theoretical and methodological advantages.

Firstly, it enables causal investigations of neuronal oscillatory phase, providing a possible conceptual advance over correlational M/EEG studies that rely on the *post-hoc* extraction of phase (Herrmann et al., 2015). Secondly, it allows sampling behavior at any desired pre-defined oscillatory phase across a pre-defined and balanced number of trials. Thirdly, the ability to modulate, rather than merely observe, neuronal oscillations with tACS enables to establish and maintain high levels of neuronal oscillatory activity across the trials of an experiment, which was previously shown to facilitate phase investigations (Mathewson et al., 2010). Fourthly, while M/EEG studies of neuronal oscillatory phase typically focus on frequency bands found to exhibit high-level oscillatory activity, the tACS approach enables modulation of oscillations in any desired frequency band. This enables robustly controlled studies including pre-defined control frequencies and control phases. Finally, M/EEG-based approaches cannot adaptively change target stimulus properties dependent on oscillatory phase during recording (because phase conditions per trial are unknown *a priori*). This can be circumvented with the tACS phase-based stimulus presentation approach. Specifically, our setup makes it possible to conduct psychophysical staircase procedures, for multiple phase conditions in parallel: thresholds (e.g., sensory or TMS) can be obtained for each desired oscillatory phase separately, even in fully interleaved and/or multi-modal paradigms. This level of control over both oscillatory phase and multi-modal stimulus events therefore allows great flexibility, increased statistical power and experimental control, but also unique opportunities that conventional M/EEG experiments cannot implement, exemplified by staircase procedures.

To conclude, while tACS as an entrainment method to modulate neuronal oscillatory power is well-established, the approach presented here flexibly extends its capabilities to study the causal role of oscillatory phase. Bringing phase-based stimulus presentation under full experimental control opens up a range of new applications that we encourage the community to consider.

## Author contributions

SO, CB, JR, and LR developed the software and hardware solutions. SO and TG performed the validation measurements and analyses. SO, TG, AS, and LR wrote the manuscript.

## Funding

This work was supported by the Netherlands Organization for Scientific Research (Veni grant 451-11-014 to LR, 451-13-024 to TG, Toptalent grant 406-11-068 to SO, VICI grant 453-15-008 to AS).

### Conflict of interest statement

The authors declare that the research was conducted in the absence of any commercial or financial relationships that could be construed as a potential conflict of interest.
