# Supplementary material for: Stimulus Presentation at Specific Neuronal Oscillatory Phases Experimentally Controlled with tACS: Implementation and Applications
Source: Front Cell Neurosci. 2016 Oct 18;10:240. doi: 10.3389/fncel.2016.00240 (PMC5067922; doi:10.3389/fncel.2016.00240)
Supplement: Supplementary file 1 [file Table1.DOCX]

| **Supplementary table 1. Absolute Phase Shift** | | | | | | | | | | |
| --- | --- | --- | --- | --- | --- | --- | --- | --- | --- | --- |
|  | Phase bin 1 | | Phase bin 2 | | Phase bin 3 | | Phase bin 4 | | Phase bin 5 | |
|  | deg | ms | deg | ms | deg | ms | deg | ms | deg | ms |
|  | |  |  |  |  |  |  |  |  |  |
| Experiment 1 | |  |  |  |  |  |  |  |  |  |
| 5 Hz | 1.03 | 0.57 | 0.88 | 0.49 | 0.95 | 0.53 | 0.99 | 0.55 | 1.34 | 0.74 |
| 10 Hz | 1.93 | 0.54 | 1.86 | 0.52 | 1.97 | 0.55 | 2.21 | 0.61 | 1.45 | 0.40 |
| 20 Hz | 3.75 | 0.52 | 3.77 | 0.52 | 4.07 | 0.56 | 3.81 | 0.53 | 3.77 | 0.52 |
| 40 Hz | 7.64 | 0.53 | 5.72 | 0.40 | 7.54 | 0.52 | 7.44 | 0.52 | 7.67 | 0.53 |
| 80 Hz | 15.03 | 0.52 | 15.07 | 0.52 | 14.92 | 0.52 | 14.88 | 0.52 | 14.59 | 0.51 |
|  | |  |  |  |  |  |  |  |  |  |
| Experiment 2 | |  |  |  |  |  |  |  |  |  |
| 5 Hz | 1.85 | 1.03 | 1.73 | 0.96 | 1.82 | 1.01 | 1.84 | 1.02 | 2.20 | 1.22 |
| 10 Hz | 3.45 | 0.96 | 3.44 | 0.96 | 3.54 | 0.98 | 3.79 | 1.05 | 3.02 | 0.84 |
| 20 Hz | 6.80 | 0.95 | 6.66 | 0.92 | 6.30 | 0.87 | 6.69 | 0.93 | 6.68 | 0.93 |
| 40 Hz | 13.29 | 0.92 | 13.19 | 0.92 | 13.20 | 0.92 | 12.99 | 0.90 | 13.08 | 0.91 |
| 80 Hz | 26.06 | 0.90 | 25.55 | 0.89 | 25.46 | 0.88 | 25.25 | 0.88 | 26.40 | 0.92 |
|  | | | | | | | | | | |
| For a description of the table, see Table 1 in manuscript. | | | | | | | | | | |
