# Supplementary material for: Stimulus Presentation at Specific Neuronal Oscillatory Phases Experimentally Controlled with tACS: Implementation and Applications
Source: Front Cell Neurosci. 2016 Oct 18;10:240. doi: 10.3389/fncel.2016.00240 (PMC5067922; doi:10.3389/fncel.2016.00240)
Supplement: Supplementary file 2 [file Table2.DOCX]

| **Supplementary table 2. Phase Consistency** | | | | | | | | | | |
| --- | --- | --- | --- | --- | --- | --- | --- | --- | --- | --- |
|  | Phase bin 1 | | Phase bin 2 | | Phase bin 3 | | Phase bin 4 | | Phase bin 5 | |
|  | maximum offset | | maximum offset | | maximum offset | | maximum offset | | maximum offset | |
|  | deg | ms | deg | ms | deg | ms | deg | ms | deg | ms |
|  | |  |  |  |  |  |  |  |  |  |
| Experiment 1 | |  |  |  |  |  |  |  |  |  |
| 5 Hz | 0.22 | 0.12 | 0.73 | 0.41 | 0.19 | 0.10 | 0.55 | 0.31 | 14.31 | 7.95 |
| 10 Hz | 0.37 | 0.10 | 0.37 | 0.10 | 2.08 | 0.58 | 12.76 | 3.55 | 16.65 | 4.63 |
| 20 Hz | 0.77 | 0.11 | 0.71 | 0.10 | 172.73 | 23.99 | 3.96 | 0.55 | 0.71 | 0.10 |
| 40 Hz | 1.53 | 0.11 | 55.71 | 3.87 | 1.40 | 0.10 | 1.54 | 0.11 | 1.60 | 0.11 |
| 80 Hz | 2.97 | 0.10 | 2.93 | 0.10 | 2.69 | 0.09 | 2.96 | 0.10 | 2.55 | 0.09 |
|  | |  |  |  |  |  |  |  |  |  |
| Experiment 2 | |  |  |  |  |  |  |  |  |  |
| 5 Hz | 0.37 | 0.21 | 1.15 | 0.64 | 0.30 | 0.17 | 0.43 | 0.24 | 14.11 | 7.84 |
| 10 Hz | 0.50 | 0.14 | 0.63 | 0.17 | 1.25 | 0.35 | 13.42 | 3.73 | 15.78 | 4.38 |
| 20 Hz | 1.03 | 0.14 | 0.78 | 0.11 | 15.54 | 2.16 | 3.88 | 0.54 | 0.70 | 0.10 |
| 40 Hz | 1.50 | 0.10 | 3.10 | 0.22 | 1.52 | 0.11 | 1.70 | 0.12 | 1.75 | 0.12 |
| 80 Hz | 3.46 | 0.12 | 3.24 | 0.11 | 2.71 | 0.09 | 4.56 | 0.16 | 3.49 | 0.12 |
|  |  |  |  |  |  |  |  |  |  |  |
|  | Phase bin 1 | | Phase bin 2 | | Phase bin 3 | | Phase bin 4 | | Phase bin 5 | |
|  | 95th percentile  offset | | 95th percentile offset | | 95th  percentile offset | | 95th percentile offset | | 95th  percentile offset | |
|  | deg | ms | deg | ms | deg | ms | deg | ms | deg | ms |
|  | |  |  |  |  |  |  |  |  |  |
| Experiment 1 | |  |  |  |  |  |  |  |  |  |
| 5 Hz | 0.18 | 0.10 | 0.36 | 0.20 | 0.18 | 0.10 | 0.15 | 0.08 | 0.53 | 0.29 |
| 10 Hz | 0.33 | 0.09 | 0.34 | 0.10 | 0.41 | 0.11 | 0.64 | 0.18 | 0.87 | 0.24 |
| 20 Hz | 0.68 | 0.09 | 0.66 | 0.09 | 6.63 | 0.92 | 0.71 | 0.10 | 0.66 | 0.09 |
| 40 Hz | 1.44 | 0.10 | 5.11 | 0.35 | 1.33 | 0.09 | 1.46 | 0.10 | 1.31 | 0.09 |
| 80 Hz | 2.72 | 0.09 | 2.70 | 0.09 | 2.51 | 0.09 | 2.59 | 0.09 | 2.43 | 0.08 |
|  | |  |  |  |  |  |  |  |  |  |
| Experiment 2 | |  |  |  |  |  |  |  |  |  |
| 5 Hz | 0.32 | 0.18 | 0.38 | 0.21 | 0.28 | 0.15 | 0.29 | 0.16 | 0.74 | 0.41 |
| 10 Hz | 0.40 | 0.11 | 0.49 | 0.14 | 0.56 | 0.16 | 0.70 | 0.19 | 0.94 | 0.26 |
| 20 Hz | 0.81 | 0.11 | 0.70 | 0.10 | 1.80 | 0.25 | 0.91 | 0.13 | 0.61 | 0.09 |
| 40 Hz | 1.36 | 0.09 | 1.38 | 0.10 | 1.38 | 0.10 | 1.39 | 0.10 | 1.44 | 0.10 |
| 80 Hz | 2.65 | 0.09 | 2.96 | 0.10 | 2.64 | 0.09 | 2.97 | 0.10 | 3.10 | 0.11 |
|  | | | | | | | | | | |
| For a description of the table, see Table 2 in manuscript. Note the similarity with DataStreamer but the large size of maximum offsets. | | | | | | | | | | |
