# Supplementary material for: Stimulus Presentation at Specific Neuronal Oscillatory Phases Experimentally Controlled with tACS: Implementation and Applications
Source: Front Cell Neurosci. 2016 Oct 18;10:240. doi: 10.3389/fncel.2016.00240 (PMC5067922; doi:10.3389/fncel.2016.00240)
Supplement: Supplementary file 3 [file Table3.DOCX]

| **Supplementary table 3. Overview: results collapsed over phase bins** | | | | | | |
| --- | --- | --- | --- | --- | --- | --- |
|  | Absolute phase offset | | Phase Consistency: full | | Phase Consistency: 95th % | |
|  | deg | ms | deg | ms | deg | ms |
|  | | |  | |  | |
| Experiment 1 | | |  |  |  |  |
| 5 Hz | 1.04 | 0.58 | 14.31 | 7.95 | 0.47 | 0.26 |
| 10 Hz | 1.88 | 0.52 | 16.65 | 4.63 | 0.66 | 0.18 |
| 20 Hz | 3.84 | 0.53 | 172.73 | 23.99 | 0.74 | 0.10 |
| 40 Hz | 7.20 | 0.50 | 55.71 | 3.87 | 2.77 | 0.19 |
| 80 Hz | 14.90 | 0.52 | 2.97 | 0.10 | 2.64 | 0.09 |
|  | | |  |  |  |  |
| Experiment 2 | | |  |  |  |  |
| 5 Hz | 1.89 | 1.05 | 14.11 | 7.84 | 0.47 | 0.26 |
| 10 Hz | 3.45 | 0.96 | 15.78 | 4.38 | 0.67 | 0.19 |
| 20 Hz | 6.63 | 0.92 | 15.54 | 2.16 | 0.92 | 0.13 |
| 40 Hz | 13.15 | 0.91 | 3.10 | 0.22 | 1.39 | 0.10 |
| 80 Hz | 25.74 | 0.89 | 4.56 | 0.16 | 2.71 | 0.09 |
|  |  |  |  |  |  |  |
| For a description of the table, see Table 3 in manuscript. Note the similarity with DataStreamer but the large size of maximum offsets. | | | | | | |
