# Supplementary figures and images for: Stimulus Presentation at Specific Neuronal Oscillatory Phases Experimentally Controlled with tACS: Implementation and Applications
Source: Front Cell Neurosci. 2016 Oct 18;10:240. doi: 10.3389/fncel.2016.00240 (PMC5067922; doi:10.3389/fncel.2016.00240)

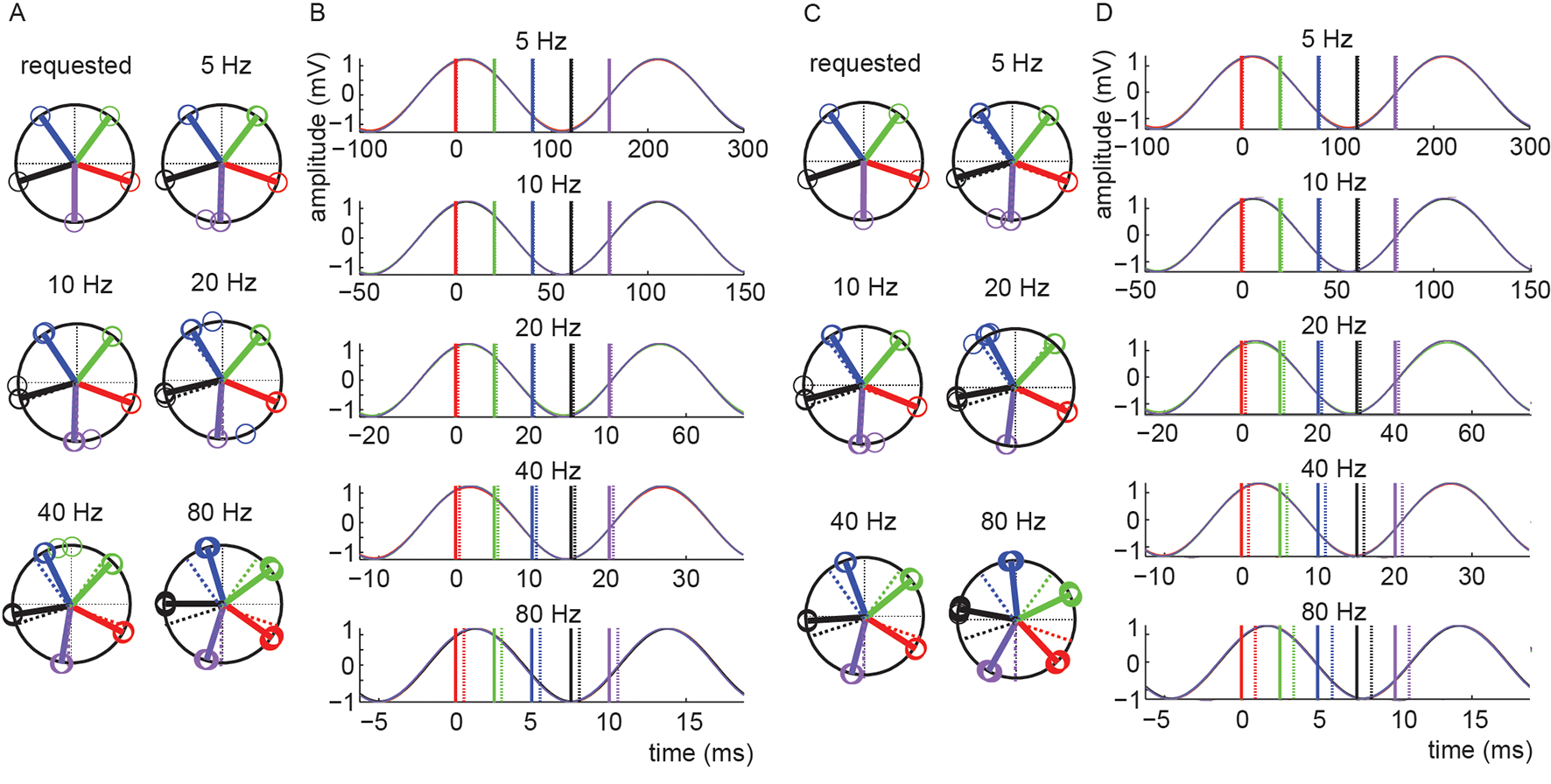

Supplement: Supplementary file 4 [file Image1.TIF]
